# A Review of the Impact of Occupational Contact Dermatitis on Quality of Life

**DOI:** 10.1155/2011/964509

**Published:** 2011-03-16

**Authors:** Melisa Yi Zhi Lau, John Anthony Burgess, Rosemary Nixon, Shyamali C. Dharmage, Melanie Claire Matheson

**Affiliations:** ^1^Centre for Molecular, Environmental, Genetic & Analytic (MEGA) Epidemiology, School of Population Health, The University of Melbourne, Level 1, 723 Swanston Street, Carlton, VIC 3053, Australia; ^2^Occupational Dermatology Research and Education Centre, Skin and Cancer Foundation Inc., Level 1, 80 Drummond Street, Carlton, VIC 3053, Australia

## Abstract

Occupational contact dermatitis (OCD) is the most common occupational skin disease in many countries. We reviewed the current evidence on how OCD impacts on quality of life (QoL). The three commonly used QoL questionnaires in OCD were the Short-Form Health Survey (SF-36), the Dermatology Life Quality Index (DLQI), and the Skindex. Despite the availability of a variety of validated QoL instruments, none of them is specific to OCD or entirely adequate in capturing the impact of OCD on QoL. Nonetheless, the results of this paper do suggest a significant impact. Use of QoL measures in clinical settings will provide patients with an opportunity to express their concerns and assist clinicians to evaluate the effectiveness of management beyond the clinical outcomes. This paper also highlights the lack of a disease-specific QOL instrument and the importance of developing a validated measure to assess QOL in OCD, enabling comparison across countries and occupational groups.

## 1. Introduction

Occupational contact dermatitis (OCD) has been defined as a pathologic condition of the skin for which occupational exposure can be shown to be a major cause or contributory factor [1]. OCD is relatively common and in many countries, OCD accounts for the majority of cases of occupational diseases [2]. In Australia, the incidence of OCD as reported in 2005 was 2.15 per 10,000 full-time workers per year [3] and internationally, the incidence of OCD ranged from 1.3 to 8.1 per 10,000 full-time workers per year over the past two decades [4].

The two most common types of OCD are allergic contact dermatitis (ACD) and irritant contact dermatitis (ICD). ACD is a delayed-type immunological reaction in response to cutaneous contact with an allergen in sensitised individuals and is diagnosed by patch testing, while ICD results from direct contact with aggravating factors such as wet work, soap, solvents, and heat that triggers the release of inflammatory mediators [2]. No routine clinical testing is available; therefore, the diagnosis is often made by exclusion of allergy in ICD. In the acute condition, the OCD may manifest as itching, redness, scaling, vesiculation, and clustered papulovesicles while fissuring, hyperkeratosis, and lichenification occur in the more chronic state [2].

The impact of OCD is often underestimated because the course of the disease is not life-threatening and minor degrees of OCD are accepted as “part of the job”. However, OCD can have profound effects involving the need to change occupation and take prolonged sick leave, as well as limiting leisure activities, interfering with the ability to perform household chores and the necessity to pursue time-consuming treatment [5]. These all affect the quality of life (QoL). 

The prognosis of OCD is poor with almost half of all patients having a condition that does not fully resolve and continues to have an impact on QoL [6, 7]. The aim of this paper is to evaluate and summarise the current evidence of the impact of OCD on QoL and examine the evidence on the sensitivity of the current measures of QoL in OCD.

## 2. Quality of Life Measures in OCD

Over recent years, much emphasis has been placed on measuring the impact of skin diseases on QoL [17]. There has been a change in emphasis away from the traditional clinician-determined method of assessing OCD severity to using the patient's perspective, as assessed by QoL scores [25].

The use of QoL measures provides patients with an opportunity to express their concerns and assist clinicians in their evaluation of the overall effectiveness of management [17]. Generic questionnaires are used to estimate the patient's general QoL in various clinical settings while disease-specific questionnaires concentrate on a particular disease. Dermatology-specific questionnaires describe the effects of particular skin conditions and allow comparison of the impacts of different skin conditions [26].  Table 1 lists some of the many generic [8–16] and dermatology-specific [17–24] questionnaires that are commonly used in dermatology.

The Short Form Health Survey (SF-36), the most studied and validated QoL instrument, is the predominantly used generic instrument in dermatology [27]. It can be used across different study populations, allowing comparison between diseases [26]. The SF-36 assesses QoL in eight domains: physical functioning, role limitation as a result of physical functioning, bodily pain, general health, vitality, social functioning, role limitation because of emotional difficulties, and mental health, as well as a question about health transition. Along with the eight domains of the SF-36, there are also two summary measures of physical and mental health called the physical component summary (PCS) score and the mental component summary (MCS) score. However, the PCS of SF-36 was found to be less relevant to dermatology patients than the MCS [28]. Other generic QoL instruments have been used infrequently in dermatology over the past decade.

Since its development in 1994, the Dermatology Life Quality Index (DLQI) questionnaire has been used widely in assessing QoL in patients with a skin disease [18]. It consists of ten questions exploring the effect of the skin condition on six domains: symptoms and feelings, daily activities, leisure, work, personal relationships, and treatment. Compared to other dermatology-specific QoL instruments, it is easy to use and has fewer questions, which might reduce the number of missing responses. Although the DLQI has been found to be a valid measure of QoL in dermatology, it may not be very sensitive in detecting small impairments [27].

Skindex, another dermatology-specific instrument, has also been shown to be valid, reliable, and responsive in assessing QoL in dermatology patients [21]. The Skindex measures QoL primarily in three domains: emotions, symptoms, and functioning. Different versions of Skindex can be used depending on the clinical setting. However, interpretation of scores may be difficult as the meaning of the scores is not well documented [27]. A combination of both generic and dermatology-specific QoL instruments has been suggested by previous reviewers [5, 27] as the best way to fully capture the effect of dermatological conditions on QoL.

## 3. Characteristics of Studies on QOL and OCD

We identified 11 studies that have investigated QoL in OCD [29–39] and these are summarised in Table 2. The study design and data collection methods varied greatly across these studies. Seven were follow-up studies [29, 31, 32, 34–36, 39], of which five were performed retrospectively [29, 31, 34, 35, 39], while four studies were cross-sectional [30, 33, 37, 38]. The study participants were either recruited from dermatology clinics [29–33, 35, 39] or national registries [34, 36–38] and the study samples ranged in size from 36 to 560 people. Although the participants in all studies had a clinical diagnosis made at first contact, in only 8 studies were further investigations done to confirm the clinical diagnosis [30–32, 34–37, 39]. Seven of the 11 studies reviewed reported the percentage of participants with atopy, with the figure ranging from 7.1% to 92.4% of the study participants [30, 31, 34, 35, 37–39]. Five studies used the SF-36 or part thereof [29, 33, 37–39], seven used the DLQI [29, 30, 32, 33, 35, 36, 39], and three used the Skindex questionnaire [31, 37, 38]. Eight studies used the combination of a dermatology-specific instrument and either a commonly used generic instrument or a self-developed questionnaire [29–31, 33, 35, 37–39]. One study used a self-developed descriptive questionnaire as the only QOL instrument [34].

## 4. Results

Overall the studies reviewed have shown a reduction in QoL in patients with OCD (Table 2). The mean overall SF-36 score was reported in two studies [33, 39]. The aggregated score of the PCS in these studies were 45.3 [33] and 52 [39] while the aggregated MCS scores were 46.4 [33] and 51 [39]. The score of the average overall quality of life for the general population is 50 [40], thus both physical and mental components summary scores in patients with OCD were slightly lower than those found in the general population. Two other studies reported the individual domain scores of the SF-36 rather than the summary score [37, 38]. The vitality domain was the most affected or had the lowest scores in three studies [37–39] while one study found that role limitations as a result of physical difficulty were the most affected domain [33].

Of the seven studies that used DLQI to assess QoL, five reported an overall mean score [29, 32, 33, 36, 39]. One study reported a small effect of OCD on QoL [39], three found a moderate effect [29, 33, 36], and one reported a large effect on QoL [32]. The most affected domains in the DLQI were symptoms and feelings [29, 30, 33, 36, 39] and work [29, 32, 33, 35]. The large variation may relate to different methods of patient selection, such as from referral clinics and workplaces, and duration of followup.

Two out of the three studies that used the Skindex reported the individual domain scores [31, 37]. The domain with the highest score in the Soder et al. [37] study was symptoms (score: 56.9), while Kadyk et al. [31] reported that the emotions domain had the highest score (score: 33.8) when compared to other domains in the questionnaire. Although a higher score generally indicates a greater effect on QoL, the degree of impairment could not be estimated due to absence of reference values or population norm score [40]. 

The different QoL measures all assess the impact of disease on various domains including symptoms/feelings, effect on work, daily activities and functioning, social/interpersonal activities, and other aspects of QoL. The effects of OCD on these different aspects of quality of life are summarised in Table 3.

### 4.1. Symptoms and Feelings

OCD frequently invokes strong negative emotions such as frustration, embarrassment, and depression, a reflection that the skin is responsible in large part for an individual's presentation [41]. These negative psychosocial effects were strongly associated with a low or reduced QoL [31, 36]. Symptoms and feelings were the most affected domains of QoL in nine out of the 11 studies [29, 30, 33–39]. In the DLQI, this domain captures information on symptoms such as itch, sore/pain and stinging, and feelings of embarrassment and self-consciousness over the last week. All three studies that reported individual DLQI domain scores found a very large impact of OCD on symptoms and feelings domain [29, 33, 39]. In one of the reviewed studies, 61% of participants complained of symptoms and 36% felt embarrassed about their OCD [30].

The Skindex assesses the symptoms and feelings in two individual domains [21]. Similar to the DLQI, the Skindex captures data on itch, stinging or burning, pain and irritation, as well as emotions like feeling worried, bothered by appearance, being frustrated, embarrassed, and feeling depressed about the skin. One study using Skindex found the symptom domain had the highest reported average score followed by the emotions domain [37] while another study found no significant difference in these two domains when a comparison was made between those with and without occupational ACD [31].

The mental health domain from the SF-36 corresponds to the symptoms and feelings domain in the DLQI and the Skindex. However, its emphasis is more on emotions rather than symptoms. The mental health domain includes questions about feeling nervousness, upset, depressed, calm and peaceful, and happy. The scores were significantly reduced, indicating a negative effect of OCD on mental health, in three studies using the SF-36 [33, 37, 38].

### 4.2. Work

The impact of OCD is most frequently reported in terms of its effect on work activities. In the SF-36, the effect on work is measured by the domain role limitation caused by physical functioning over the past four weeks. The domain measures the limitation of performing tasks, such as cutting down the amount of time spent on work, accomplishing less work than expected, limitation in the kind of work, and difficulties in performing work. Of the five studies that used the SF-36, two reported significantly reduced domain scores when compared to population norms [33, 38] while another two had no difference with the population norms scores [37, 39]. One study did not report the domain score [29] but found that 45% of males accomplished less at work and 40% of females had difficulty in performing work.

Seven studies were using the DLQI which measured the impact of OCD on work impediment and predicament at work over the last week. Only three studies reported the scores [29, 33, 39]. Two found a moderate effect of OCD on work [29, 39] while one found a very large impact on work [33]. Other studies found that 20% of their participants could not work [30], 43% had interference with work [30], and 39% reported severe impact on work [32].

In those with OCD, the proportion that had either changed job or made job modifications ranged between 23.1%–82% [34, 35, 37]. An explanation for this large variation may be due to the variety of occupations represented in this paper or due to differences in disease duration between studies. The high proportion of people with job modification in the study by Meding et al. [34] might have been due to the use of nonstandardised questions compared to other validated instruments.

### 4.3. Daily Activities/Physical Functioning

Six studies documented the effect of OCD on daily activities or physical functioning using the DLQI, Skindex, and/or the SF-36. The SF-36 has ten questions about the impact on daily physical functioning such as bathing or dressing, walking, and climbing stairs. When the domain scores were compared to the general population scores, one study found a significant impairment in the physical functioning domain [33], whereas in two other studies, the authors found no effect on physical functioning in OCD patients [38, 39].

The daily activities domain is assessed by two questions in the DLQI: interference with housework, shopping or gardening, and influence to the choice of clothing over the last week. Two studies using the DLQI, found a moderate effect on the daily activities domain [33, 39]. Two other studies that used the DLQI but did not document the scores reported that 23% [30] and 45.7% [35] of participants had interference in this domain. Holness [30] replaced one question from the DLQI with one related to sleep disturbance and 44% of patients reported an effect of OCD on sleep. A similar impact on daily activities was reported by Kadyk et al. [31], who found that OCD had a significant impact on daily activities in those with occupational ACD compared to ACD that was not occupational. This study used the Skindex questionnaire which did not further define daily activities. Although most studies reported interference with daily activities or physical functioning, it was often the least affected domain.

### 4.4. Social/Interpersonal Relationships

OCD can have a considerable impact on social or interpersonal relationships and this domain is assessed by the social functioning domain in the SF-36 and personal relationships domain in the DLQI. The SF-36 assesses this domain by inquiring about the frequency and extent of interference with normal social activities such as visiting friends and relatives. Although this domain score was significantly lower than population norms in two studies [33, 38], OCD was not shown to have an effect in interpersonal relationships in one study [39].

Conversely, the DLQI identifies the effect of OCD on sexual difficulties and problems with partners, close friends, or relatives, and three of the reviewed studies found no or little effect on this domain [29, 33, 39]. Some studies reported the proportion instead of domain scores and in these studies, the percentage of OCD patient affected by OCD on their interpersonal relationships ranged from 19 to 44% [30, 32, 35]. Other studies also found that those with occupational ACD were concerned about interacting with coworkers because of the skin disease [31], while 18.6% reported difficulty in family relationships, including rejection by a spouse and in some cases, divorce [35].

### 4.5. Effects on Gender, Age, and Disease Severity

A few studies have found that the effect of OCD on QoL varied between males and females. Several studies reported a higher impact of OCD on QoL in females [33, 37], while others reported similar effects in men and women [29, 36, 39]. The apparent greater effect in females may be explained by a higher prevalence of health seeking behaviour by females in general [42], or a larger effect on the mental and social QoL domain in females [29, 34, 38]. Some studies detected a sex difference in QoL using a generic measure [33, 37] while other studies detected a difference when a dermatology-specific instrument was used [38].

Increasing age had been found by one study to increase the impact of OCD on QoL [37] but this was contradicted by three other studies [29, 33, 36] that found no significant association between age and QoL in patients with OCD. In a Swedish study, a significant interaction between both total PCS and the physical function domain of SF-36 and age was found in females but not males [33]. In a previous paper, the association between age and OCD disappeared when occupation was taken into account in the analysis [2]; therefore, it seemed that age was unlikely to have a major influence on QoL in OCD patients.

Of the included studies, five assessed the severity of OCD [35–39]. A high degree of disease severity was generally associated with a low QoL [36, 38, 39]. No significant correlation was found between severity of skin disease and QoL as determined by SF-36 in two studies [37, 39]. In contrast, when a dermatology-specific instrument was used, significant correlations between OCD severity and QoL were reported [36, 37, 39]. However, the assessment of the severity of skin disease has been challenging and was not consistent across studies. Some assessments were based on more objective criteria such as morphology, extent of dermatitis, and frequency of flare-up [35, 36, 39], while others used a simple subjective description to determine severity [38, 39].

### 4.6. Prognosis

Several studies reassessed the participants on followup. The improvement rate varied widely from as little as 21% to as much as 65% [34, 37, 39]. Some participants had developed persistent postoccupational dermatitis (PPOD) [43] when reviewed [35, 39]. PPOD has been associated with a reduced ability to work, a negative impact on a financial situation or interference with personal life [44, 45].

## 5. Discussion

OCD-related QoL was found to be reduced in all studies with the areas of highest impact being effects on work and symptoms/feelings. Nevertheless, a large variation between studies was observed in design, patient recruitment, participants' characteristics, assessment of disease severity, and QoL instruments used. This variation in methodology across studies makes comparisons between studies somewhat difficult. Another difficulty in epidemiological studies of OCD is the lack of a clear and standardised definition of OCD. The definitions of OCD used in the reviewed studies have been inconsistent. OCD is not a homogeneous condition: it may be ICD or ACD or contact urticaria [2]. Apart from the criteria provided by Mathias in 1989 [46], no other studies had provided a definition for OCD. Our group has previously referred to the lack of standardisation of OCD [4]. 

The current paper raises several issues to be considered in future research. We found that several studies used both generic and dermatology-specific questionnaires to assess QOL. The main advantage of this is to fully capture the effect of OCD on QOL. Given OCD influences so many different areas of lifestyle, health, economic burden, emotions, and feelings, it is unlikely that the use of either a generic or dermatology-specific instrument will be entirely adequate. To date, there is no validated QoL instrument specific to OCD and the development of a simple yet comprehensive QoL instrument specific to OCD would be of considerable clinical benefit. Currently an international group is investigating the possibility of adding OCD-specific questions to the DLQI and one of the coauthors (RN) of this paper is currently part of a project that is piloting the new questionnaire (personal communication, Dr Päivikki Susitaival).

One important area of research missing from the current literature on QOL in OCD is the evaluation of QoL over time. The assessment of QOL at baseline and then at followup allows the change in physical symptoms and the other components of QOL to be assessed in a validated, consistent manner. Using QoL measures in routine clinical settings will provide patients with an opportunity to express their concerns and assist clinicians in evaluating the effectiveness of management beyond the clinical outcomes. Among the studies included in this paper, only two [32, 36] evaluated QoL over time and found an improvement in QOL.

Data on the impact of disease severity on QoL is limited and even the existing literature has assessed disease severity inconsistently across studies, if at all. Furthermore, both objective and subjective measures have been used to evaluate disease severity making interpretation and comparison even more difficult. OCD is primarily a disease of the hands and the existing and validated measures of disease severity [47, 48] rely heavily on body surface involvement. Severity scales have been developed for use by the physician in the assessment of hand eczema [49–51] and one has been developed by our group specifically to assess functional limitations as well as severity in OCD [52, 53]. Using these objective measures of severity in future research would allow for direct comparisons across studies in different countries and occupational groups.

In conclusion, this paper has shown that OCD has a heavy impact on QOL, particularly on work arrangements, which can have important financial and social consequences if not addressed adequately. We would highlight the opportunity for more routine use of QoL measures in patient management. Further studies would benefit from using multiple validated instruments to assess both QOL and disease severity, and specifically a comprehensive QoL instrument specific to OCD, which would enable comparison across countries and occupational groups.

## Figures and Tables

**Table 1:** Generic and dermatology-specific quality of life instruments used in occupational contact dermatitis.

Instrument	Number of questions	Domains	Scoring
*Generic QoL instrument*			
SF-36 [8–10]	36	(i) Physical functioning	Each domain is transformed into a score of 0–100. The scores are then summarised into mental (MCS) and physical components (PCS). Higher score indicates less impaired QoL.
		(ii) Role limitations due to physical difficulty	
		(iii) Bodily pain	
		(iv) General health	
		(v) Vitality	
		(vi) Social functioning	
		(vii) Role limitations due to emotion	
		(viii) Mental health	
		(ix) Health transition	
			
NHP [11, 12]	38	(i) Energy level	Positive responses in a domain are summed up or weighting items to calculate a score ranging from 0–100.
		(ii) Emotional reactions	
		(iii) Physical mobility	
		(iv) Pain	
		(v) Social isolation	
		(vi) Sleep	
			
SIP [13]	136	(i) Physical	Scores are calculated per scale, domain, and as an overall score ranging from 0–100.
		(ii) Psychosocial	
		(iii) Independent categories	
			
GHQ [14, 15]	28	(i) Somatic symptoms	A total score (range: 0–84) or a domain score is obtained by summing up each item.
		(ii) Anxiety and insomnia	
		(iii) Social dysfunction	
		(iv) Severe depression	
			
DUKE [16]	17	(i) Health	Score from individual item is summed and transformed to obtain a total score ranging from 0–100. Higher score indicates less impairment to QoL.
		(ii) Dysfunction	

*Dermatology-specific QoL instrument*			
DLQI [17, 18]	10	(i) Symptoms and feelings	A total score between 0–30 is obtained by summing the score of each question. Lower score indicates less impaired QoL.
		(ii) Daily activities	
		(iii) Leisure	
		(iv) Work/school	
		(v) Personal relationships	
		(vi) Treatment	
			
Skindex [19–21]	16, 17, 29, 61	(i) Emotions	Each domain is scored individually with a possible score ranging from 0–100. Lower score indicates less impaired QoL.
		(ii) Functioning	
		(iii) Symptoms	
			
DSQL [22, 23]	52	(i) Psychosocial	A summary score was calculated by adding all raw scores from each item.
		(ii) Activities	
		(iii) Symptoms	
			
DQOLS [24]	41	(i) Physical symptoms	The score for each domain is calculated separately, transforming to a score ranging from 0–100.
		(ii) Daily activities	
		(iii) Social activities	
		(iv) Work/school experience	
		(v) Self perception	
		(vi) SF-36 vitality subscale	
		(vii) SF-36 mental health subscale	

SF-36: Short-Form Health Survey; NHP: Nottingham Health Profile; SIP: Sickness Impact Profile; WHOQOL: World Health Organisation Quality of Life; GHQ: General Health Questionnaire; DUKE: Duke Health Profile; DLQI: Dermatology Life Quality Index; DSQL: Dermatology-specific Quality of Life; DQOLS: Dermatology Quality of Life Scales.

**Table 2:** Study design and characteristics of different studies on OCD.

Author	Country and year	Study design	Recruitment	No. of participants (% of female)	Atopy (%)	Mean age ± s.d., range	Definition of diagnosis	Investigations
Hutchings et al. [29]	UK, 2001	Retrospective followup using postal survey	Contact dermatitis clinic	181 (47.0)	NA	18–67	OCD diagnosed by physician at baseline	NA

Holness [30]	Canada, 2001	Cross-sectional	Occupational Health Clinic	339 (54)	59	42.5	Contact dermatitis diagnosed by physician	Patch-testing

Kadyk et al. [31]	USA, 2003	Retrospective followup by postal survey	Contact dermatitis test centre	149 (64.5)	11.9	53.0 ± 16.6	ACD diagnosed by physician at baseline	Patch-testing at baseline

Lewis et al. [32]	UK, 2004	Followup	Contact dermatitis clinic	36	NA	36 (22–56)	Work-related natural latex allergy diagnosed by physician	Prick test at baseline

Wallenhammar et al. [33]	Sweden, 2004	Cross-sectional	Occupational and Environmental Dermatology Clinic	100 (51)	NA	Females 41 (20–64), males 42 (19–64)	Hand eczema diagnosed by physician	NA

Meding et al. [34]	Sweden, 2005	Retrospective followup using postal and telephone survey	Cases from social insurance office	517 (394 postal & 123 telephone) (62.5)	24.5	16–64	Occupational skin disease diagnosed by physician at baseline	Patch-testing at baseline

Lazarov et al. [35]	Israel, 2006	Retrospective followup by telephone survey	Contact dermatitis clinic	70 (35.7)	7.1	25–59	OCD diagnosed by dermatologist at baseline	Patch-testing at baseline

Cvetkovski et al. [36]	Denmark, 2006	Followup by mail 1 year after baseline questionnaire was returned	Danish National Board of Industrial Injuries (DNBII)	564	NA	35.8	Occupational hand eczema diagnosed by dermatologist at baseline	Patch-testing, additional prick test when relevant

Soder et al. [37]	Germany, 2007	Cross-sectional	Cleaning and kitchen employee who reported to BGW	212 (84.0)	47.2% atopic skin diathesis, 15.6% flexural eczema	41.6 ± 10.8	Employees suspected with occupational skin disease	Allergy test prior to study

Matterne et al. [38]	Germany, 2009	Cross-sectional	Healthcare workers who reported to BGW	278 (92.4)	55.4% atopy, 23.7% flexural eczema	36.9 ± 11.6	Employees suspected with occupational skin disease	NA

Lau et al. [39]	Australia, 2010	Interviewer-administered retrospective followup	Occupational dermatology clinic	113 (47.8)	48.7	41.2 ± 13.4	OCD diagnosed by dermatologist at baseline and at followup	Patch testing at baseline

NA: information not available/not performed, SIP: secondary individual prevention, BGW: Accident Prevention and Insurance Association.

**Table 3:** Effects of occupational contact dermatitis on different aspects of quality of life.

Author	Questionnaire(s) used	Mean score	Severity assessment	Overall effect on gender	Symptoms/feelings	Work	Daily activities/functioning	Social/interpersonal activities	Others
Hutchings et al. [29]	DLQI, part of SF-36	DLQI 6.6; SF-36 NA	NA	No difference	Most affected domain with higher effect on female	Affected but not prevented with higher effect on males	NA	Least affected domain	

Holness [30]	Modified DLQI, open-ended questions	DLQI NA	NA	NA	61% complained of itchiness/pain, 36% felt embarrassed	20% could not work and 43% had interference with work	32% had sleep disturbance, 23% had interference with housework/shopping	20% were affected	Those with hand involvement reported a worse QoL.

Kadyk et al. [31]	Skindex-16, additional questions on occupational impact	Skindex-16 individual scale ranges from 13.3–31.0	NA	NA	NA	Significantly more affected compared to those with nonoccupational ACD	Slightly more impaired than those with nonoccupational ACD	Concerned that interaction with coworkers may be difficult	

Lewis et al. [32]	DLQI	Before diagnosis 17.9; after diagnosis 10.9	NA	NA	NA	39% reported severe impact on work	NA	44% had issues with interpersonal relationships	

Wallenhammar et al. [33]	DLQI, SF-36	DLQI 7.4; SF-36 PCS 45.3; MCS 46.4	NA	No difference on DLQI; female had more impaired QoL on SF-36	Very large effect	Most affected domain in DLQI, significantly reduced role-physical domain in SF-36	23% females and 18% males reported interference with daily activities	Personal relationship was the least effected	

Meding et al. [34]	Self-developed questionnaires	NA	NA	NA	52% had symptoms > half of the time; females more affected, 80% reported itching to their hand eczema	82% changed work situation, 44% changed job	NA	88% reported effect on psychosocial and had to give up on their hobbies	28% had total recovery

Lazarov et al. [35]	DLQI, self-developed questionnaires	DLQI NA	NII-rated	NA	45.7% feeling shame and rejected	52.9% changed job	45.7% reported interference	18.6% had limitations in interpersonal relationship	11.4% had persistent dermatitis

Cvetkovski et al. [36]	DLQI, BDI-II	DLQI 5.5; BDI-II 7.1	DNBII-rated	No difference	Most affected domain, 9% reported moderate-to-severe depression at baseline and at followup	Most affected domain at baseline	NA	NA	Strong association between mild-to-moderate OHE and severe OHE and low QoL at baseline

Soder et al. [37]	SF-36, Skindex-29	SF-36 individual scale ranges from 53.9–81.4; Skindex-29 individual score range 23.0–56.9	Physician-assessed	Females more affected in most subscales of SF-36	Most affected domain with 12.7% reported itchiness on entire skin surface & 37.7% on hands	31.1% had <8 weeks of sick leave; 48.6% were absent from work. 23.1% from telephone survey changed or gave up their profession	Least affected domain in Skindex	NA	No significant correlation between severity of skin and QoL. 65% from telephone survey had improved skin

Matterne et al. [38]	SF-36, Skindex-29	SF-36 individual scale ranges from 54.2–89.6; Skindex-29 raw score NA	Yes	No difference on SF-36, females more affected on Skindex-29	Females reported more emotional impairment, more impaired social functioning and mental health	NA	Less impaired physical functioning compared to general population	NA	More severe skin is associated with perceived pain

Lau et al. [39]	DLQI, SF-36	DLQI 4.5; SF-36 PCS 52; MCS 51	Physician-rated, patient-rated, ODDI	No difference	Most affected domain in DLQI, lower mental health scores	Very large effect	Moderately affected as measured by DLQI, no effect in SF-36 domains	No effect on social functioning	Significant correlation between severity of skin and QoL. 21% had complete clearance; 18% progressed to PPOD

NA: information not available/not performed; DLQI: Dermatology Life Quality Index; QoL: quality of life; ACD: allergic contact dermatitis; SF-36: Short-Form Health Survey; NII: National Insurance Institute of Israel; BDI-II: Beck Depression Inventory; DNBII: Danish National Board of Industrial Injuries; OHE: occupational hand eczema; GHQ: General Health Questionnaire; SAS: Self-Acceptance Scale; PPOD: persistent postoccupational dermatitis.

## Abbreviations

SF-36: Short-Form Health Survey
NHP: Nottingham Health Profile
SIP: Sickness Impact Profile
WHOQOL: World Health Organisation Quality of Life
GHQ: General Health Questionnaire
DUKE: Duke Health Profile
DLQI: Dermatology Life Quality Index
DSQL: Dermatology-specific Quality of Life
DQOLS: Dermatology Quality of Life Scales.
